# Multidrug-Resistant *Acinetobacter baumannii* May Cause Patients to Develop Polymicrobial Bloodstream Infection

**DOI:** 10.1155/2022/8368578

**Published:** 2022-06-24

**Authors:** Qingqing Chen, Zhencang Zheng, Qingxin Shi, Huijuan Wu, Yuping Li, Cheng Zheng

**Affiliations:** ^1^Department of Critical Care Medicine, Taizhou Hospital of Zhejiang Province Affiliated to Wenzhou Medical University, Taizhou 318000, China; ^2^Health Commission of Taizhou, Taizhou 318000, Zhejiang, China; ^3^Clinical Microbiology Laboratory, Taizhou Hospital of Zhejiang Province Affiliated to Wenzhou Medical University, Taizhou 318000, China; ^4^Department of Pulmonary and Critical Care Medicine, The First Affiliated Hospital of Wenzhou Medical University, Wenzhou 325000, China; ^5^Department of Critical Care Medicine, Taizhou Municipal Hospital, Taizhou 318000, China

## Abstract

**Background:**

The incidence of polymicrobial bloodstream infections is increasing, the clinical characteristics of polymicrobial *Acinetobacter baumannii* bloodstream infections (AB-BSI) are unclear, and there are no reports of polymicrobial AB-BSI in mainland China. Therefore, our objective was to identify the clinical characteristics, risk factors, and outcomes of polymicrobial AB-BSI versus monomicrobial AB-BSI.

**Methods:**

A retrospective survey of all patients with AB-BSI from January 1, 2015, to December 31, 2019, and their clinical data were collected and analyzed by reviewing electronic medical records. All data were compared and analyzed between groups of monomicrobial and polymicrobial AB-BSI. Risk factors for polymicrobial AB-BSI were assessed using multivariable logistic regression analysis.

**Results:**

A total of 204 patients were included, of which 39 (19.1%) were patients with polymicrobial AB-BSI. The main sources of the pathogenicity of polymicrobial *Acinetobacter baumannii* bloodstream infections were skin and soft tissue (38.5% vs. 16.4%, *p*=0.002). Resistance to piperacillin/tazobactam as an independent factor for polymicrobial AB-BSI was found in multivariate analysis. Patients with polymicrobial AB-BSI had longer hospital stays compared to those with monomicrobial AB-BSI. However, there was no significant difference in mortality between the two groups.

**Conclusions:**

Polymicrobial AB-BSI accounted for a significant proportion among all AB-BSI, and it did not influence mortality but was related to slightly longer total hospital stays. Multidrug resistance was associated with the development of polymicrobial AB-BSI but does not directly lead to polymicrobial AB-BSI, whereas resistance to piperacillin/tazobactam was highly correlated with polymicrobial AB-BSI. Therefore, while treating *A. baumannii* bloodstream infections, clinicians cannot ignore the multidrug-resistant *A. baumannii*, especially piperacillin/tazobactam-resistant *A. baumannii*, which may predispose to the development of polymicrobial AB-BSI.

## 1. Introduction

Bloodstream infections (BSI) are a growing concern worldwide due to their potential consequences [1]. As one of the essential Gram-negative bacteria, *Acinetobacter baumannii* plays an essential role in hospital-acquired infection. It has been reported that *A. baumannii* BSI (AB-BSI) accounts for 9–35% of the total BSI cases [2, 3]; with the aging of the population as well as increases in intrusive operations, people have noted that the incidence rate of AB-BSI is also increasing year by year [4], and administration of broad-spectrum antibiotics has also led to a rapid increase in the drug resistance rate of *A. baumannii*, which makes multidrug-resistant (MDR) *A. baumannii* a critical threat to human health globally [5]. A recent study characterizing 39,320 *A. baumannii* isolates revealed that the prevalence of MDR *A. baumannii* had escalated from 21.4% (2003–2005) to 35.2% (2009–2012) [6]. Most BSIs are monomicrobial, but in recent years, the trend of polymicrobial BSI has been rising, accounting for about 6–34% of BSIs [7, 8]. There have been several reports on polymicrobial BSI with specific pathogens [9–12], while polymicrobial AB-BSI has not received much attention at present. Studies have shown that *A. baumannii* was associated with higher mortality in intensive care patients with bacteremia [13]. Thus theoretically, mortality of polymicrobial AB-BSI should at least be similar to that of monomicrobial AB-BSI or higher. However, we did not find any difference in mortality between polymicrobial BSI and monomicrobial BSI in those reports which focused on specific pathogens BSI [9–12]. For this reason, whether the clinical characteristics of polymicrobial AB-BSI are similar to those described in the abovementioned studies, whether multidrug-resistant bacteria lead to polymicrobial AB-BSI, whether there are differences in mortality and drug resistance rates between groups of polymicrobial AB-BSI and monomicrobial AB-BSI, and factors that are associated with polymicrobial AB-BSI are still unclear. Moreover, there was no research that focused on polymicrobial AB-BSI on the Chinese mainland. Therefore, it is necessary to analyze the clinical characteristics and risk factors of polymicrobial AB-BSI, so that clinicians can clearly understand the harm of polymicrobial AB-BSI and avoid the occurrence of polymicrobial AB-BSI in the early stage.

## 2. Materials and Methods

### 2.1. Design and Patients

This was a single-center retrospective study that collected all cases of AB-BSI from January 2015 to December 2019 at the Taizhou Hospital of the Zhejiang Province affiliated to Wenzhou Medical University. This affiliated hospital is a 2,800-bed comprehensive tertiary teaching hospital serving a broad population in the local region of Taizhou (a subtropical climate city with a population of 6 million), China. This study was conducted in accordance with the Declaration of Helsinki. The study was approved (No. K20211001) by the Ethics Committee of the Taizhou Hospital of the Zhejiang Province affiliated to Wenzhou Medical University which determined that patient consent was not required because it was a retrospective study. The criteria for inclusion in the study were patients having proven *A. baumannii* bloodstream infection and being aged 18 years or older. In this study, patients were divided into two groups according to whether microorganisms other than *A. baumannii* were isolated in the same specimen number. Age <18 years; incomplete or missing case information; *A. baumannii* considered nonpathogenic; and pregnant patients were excluded.

### 2.2. Identification of Bacterial Species and Antibiotic Susceptibility Testing

Blood culture by using the BacT/ALERT 3D system (Becton–Dickinson, Sparks, MD, USA), species identification, and antibiotic susceptibility were carried out by using the VITEK-2 (Card number: AST-GN334; AST-GP67) compact automatic microbiological analyzer (Oxoid, UK) according to the recommendations proposed by the Clinical and Laboratory Standards Institute (CLSI).

### 2.3. Definitions

Diagnosis of AB-BSI was based on CDC definitions for the bloodstream infection event^14^. We define the time at which a blood culture was collected as the onset of BSI. Isolation of one or more organisms other than *A. baumannii* from a blood culture specimen cultured with *A. baumannii* is considered a polymicrobial BSI [14]. Nosocomial BSI was defined as a positive blood culture obtained ≥48 hours after admission without evidence of infection at admission [15, 16]. Nonpathogenic bacteria were considered as contaminants, defined as one single positive blood culture in the absence of clinical manifestations [17, 18]. Appropriate antimicrobial therapy is defined as administering sensitive antibiotic therapy within 2 hours of the first culture of *A. baumannii* in the blood; administration of sensitive antibiotic therapy beyond 24 hours is considered delayed antibiotic therapy [19]. We diagnose septic shock according to Sepsis-3 [20]. MDR was defined as acquired nonsusceptibility to at least one agent in three or more antimicrobial categories [21].

### 2.4. Data Collection

The patients' data were extracted from electronic medical records. Patients' baseline characteristics included age and gender; the clinical data include underlying diseases, sequential organ failure assessment (SOFA) score, Pitt bacteremia score, Charlson Comorbidity Index (CCI) score, acute physiology and chronic health evaluation (APACHE) II score with 24 h of the onset of BSI, hospitalization wards, previous exposures, and nosocomial infection. Data on possible sources of BSI, monomicrobial/polymicrobial, and sensitivity to antibiotics were also included in our collection. All these data were collected by the same clinician to ensure the reliability of the data.

### 2.5. Statistical Analysis

Continuous variables were compared using Student's *t*-test or the Mann-Whitney *U* test, and count variables were compared using Pearson's *χ*^2^ test. Variables with a significant *p* < 0.05 level in univariate analysis were considered candidates for building stepwise logistic regression multivariate models. The two-tailed test with *p* < 0.05 was considered statistically significant. All data were statistically analyzed using SPSS 20.0 software (IBM Corp, Armonk, NY, USA).

## 3. Results

### 3.1. Demographic and Clinical Characteristics

**Figure 1:**
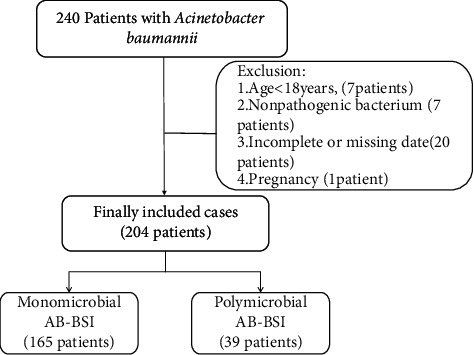
Flowchart of the study participant enrollment. Abbreviations: AB-BSI, *Acinetobacter baumannii* bloodstream infections.

A total of 240 patients with *A. Baumannii* were initially included, and 204 cases were finally recruited with 39 cases of polymicrobial AB-BSI and 165 cases of monomicrobial AB-BSI (Figure 1).  Table 1 summarizes the demographic and clinical characteristics of these patients. The median age was 65 years (IQR, 49.25–76.75), and 67.6% were male. Hypertension was the most common comorbidity (35.8%), followed by trauma (27.5%). There were no significant differences in gender or age between the two groups. A significantly high percentage of trauma or burn injuries was observed in patients with polymicrobial AB-BSI (all *p* < 0.05). Patients with polymicrobial AB-BSI had more need of blood transfusion (59% vs. 41.2%, *p*=0.045) and significant increases in urinary catheter indwelling (89.7% vs. 73.9%, *p*=0.035) compared with monomicrobial AB-BSI. It is worth noting that there is no significant difference in the severe condition presented by the APACHE II score, SOFA score, CCI, and Pitt bacteremia score between the two groups.

### 3.2. Biological Indicators

Table 2 shows the comparison of laboratory indicators between the two groups. There was no significant difference between the blood routine test, liver function indicators, and biochemical indicators between the two groups.

### 3.3. Isolates and Sources of Polymicrobial AB-BSI

**Figure 2:**
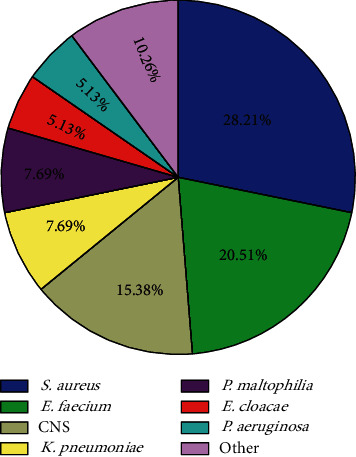
Distribution of the additional organisms in polymicrobial *Acinetobacter baumannii* bloodstream infections. Abbreviations: *S. aureus*: *Staphylococcus aureus; E. faecium*: *Enterococcus faecium; CNS*: *coagulase-negative Staphylococcus; K. pneumoniae*: *Klebsiella pneumoniae; P. maltophilia*: *Pseudomonas maltophilia; E. coli*: *Escherichia coli; P. aeruginos*a: *Pseudomonas aeruginosa*; Others: *Candida albicans* (2.6%), *Escherichia coli* (2.6%), *Candida glabrata* (2.6%) and *Morganella morganii* (2.6%).

Figure 2 shows the isolated pathogens. A total of 44 microorganisms other than *A. Baumannii* was isolated from 39 polymicrobial AB-BSI cases, with two microorganisms accounting for 87.2% and three microorganisms for 12.8%. The most common copathogen was *Staphylococcus aureus* (28.21%), followed by *Enterococcus faecium* (20.51%) and Coagulase-negative Staphylococci (15.38%).

The main source of AB-BSI was pneumonia (30.9%), followed by intra-abdominal (21.6%), and skin and soft tissue infection (20.6%) (Table 3). The sources of skin and soft tissue infections were more frequent in polymicrobial AB-BSI than monomicrobial AB-BSI (38.5% vs. 16.4%, *p*=0.002). There is no significant difference between the two in other sources.

### 3.4. Antibiotic Resistance and Appropriate Therapy

The experiment of drug sensitivity showed that tigecycline had the lowest resistance (3%), sequentially followed by ceftazidime (13.6%) and amikacin (23.9%). The resistance rate of *A. baumannii* to imipenem, cefepime, tobramycin, piperacillin/tazobactam, and ciprofloxacin was significantly higher in the polymicrobial AB-BSI group compared to the monomicrobial AB-BSI group (Table 3). The proportion of MDR *A. baumannii* in the polymicrobial group was also higher than that in the monomicrobial group, but there was no significant difference (89.7% vs. 75.8%, *p*=0.056). In addition, antibiotic treatment was delayed in 9.3% of patients within 24 hours of the release of the antibiotic sensitivity results. However, there was no statistical difference between the two groups (7.7% vs. 9.7%, *p*=0.698) (Table 1). Interestingly, we observed that the percentage of MDR *A. baumannii* decreased with the years, and the corresponding polymicrobial BSI also showed a downward trend (Supplementary Figure 1).

### 3.5. Independent Risk Factors for Polymicrobial AB-BSI

Multivariate logistic regression model analysis showed that the independent risk factor of polymicrobial AB-BSI is resistant to piperacillin/tazobactam (adjusted odds ratio (OR), 14.48; 95% confidence interval (CI), 2.07–101.24) (Table 4).

### 3.6. Outcomes

**Figure 3:**
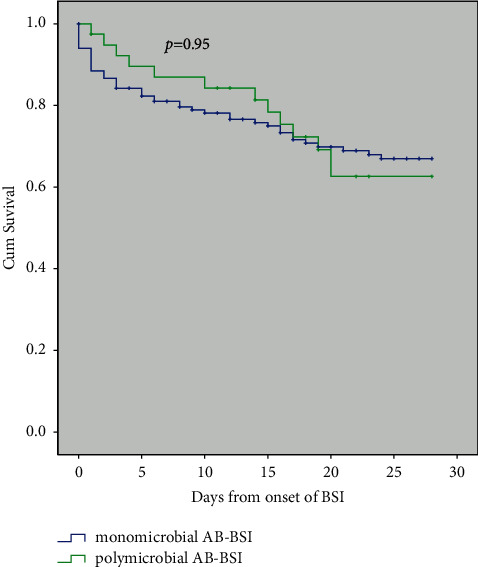
Kaplan–Meier estimates of survival in patients with polymicrobial *Acinetobacter baumannii* bloodstream infections and monomicrobial *Acinetobacter baumannii* bloodstream infections. Abbreviations: AB-BSI: *Acinetobacter baumannii* bloodstream infections.

As shown in Table 5, although there was no significant difference in length of hospital stay between the two groups, patients with polymicrobial AB-BSI appeared to have a longer hospital stay (median days, 55 (27,91) vs. 35 (18.5,81), *p* < 0.1). There were no significant differences in the 14-day, 28-day, and in-hospital mortality between the two groups (Table 5), which was consistent with the survival curves of the patients in both the groups (Figure 3).

## 4. Discussion

The main findings of our study are as follows: (1) Polymicrobial AB-BSI is not rare among *A. baumannii* bacteremia. (2) *S. aureus* was the most common copathogen in polymicrobial AB-BSI, followed by *Enterococcus faecium*. (3) MDR is more prevalent in polymicrobial AB-BSI, but is not an independent risk factor. (4) Resistance to piperacillin/tazobactam was the only independent risk factor for polymicrobial AB-BSI (Table 4). (5) Patients with polymicrobial AB-BSI might have poor outcomes than patients with monomicrobial AB-BSI, as evidenced by a longer hospital stay, but mortality did not differ significantly.

In the current study, polymicrobial AB-BSI accounted for 19.1% of *A. baumannii* BSI, which is generally consistent with previous reports of polymicrobial bacteremia, accounting for 5–20% of BSI [22–24]. Among the copathogens, *S. aureus* and *E. faecium* accounted for nearly 50% (Figure 2). Previous studies have shown that *A. baumannii* appears to be a common copathogen in polymicrobial bloodstream infections [9, 10, 25]. Methicillin-resistant *S. aureus* (MRSA) and *A. baumannii* are particularly significant in burns patients who are uniquely susceptible to infection and colonization with these organisms [26–29], whereas hospital-acquired pneumonia is primarily caused by these organisms in the intensive care unit (ICU) [30, 31]. In our study, the rather high proportion of ICU admissions, the not uncommon number of burn patients, pneumonia and skin and soft tissues as the primary source of infection, and the fact that *S. aureus* was the most important copathogen, all suggest that *A. baumannii* may have a synergistic relationship with *S. aureus* and *Enterococcus*, resulting in their common growth. Therefore, when evaluating the efficacy of various regimens for clinical outcomes of *A. baumannii*, antibiotic efficacy against concomitant isolates should also be evaluated, unless there were no concomitant isolates.

In the current study, although we found many risk factors associated with polymicrobial AB-BSI, by multivariate analysis, we found that piperacillin/tazobactam resistance was the only independent risk factor for polymicrobial AB-BSI. Although previous studies [22, 32] have demonstrated that MDR bacterial BSI is associated with polymicrobial BSI, our study also found a higher proportion of MDR in polymicrobial AB-BSI (75.8% vs. 89.7%), MDR was not an independent risk factor for polymicrobial BSI. A previous study showed that beta-lactamase-producing pathogens could provide indirect pathogenesis by protecting the other pathogens in polymicrobial infection environments [33]. We therefore speculate that, as a beta-lactamase-producing pathogen, piperacillin-resistant *A. baumannii* may shelter piperacillin/tazobactam-susceptible bacteria from piperacillin tazobactam killing in polymicrobial infection. Our study found that trauma, burns, and blood transfusions are risk factors for polymicrobial AB-BSI. Patients with trauma and burns were prone to extensive disruption of the skin barrier [34] and the presence of large numbers of blood transfusions [35], making pathogens that colonized in the skin susceptible to polymicrobial BSI via the soft tissue skin route, as well as the blood transfusion route. Thus, as one of the most common colonizing bacteria of the soft tissues of the skin, *A. baumannii* can cross the skin barrier together with other pathogens such as piperacillin/tazobactam-sensitive pathogens, thus causing polymicrobial AB-BSI. Therefore, when treating AB-BSI, clinicians should pay attention to MDR and piperacillin\tazobactam resistance, which may lead to polymicrobial AB-BSI and make treatment more difficult.

It is worth noting that we did not find any difference in mortality, including 14-day, 28-day, or in-hospital mortality between the groups of polymicrobial and monomicrobial AB-BSI, except for a slight increase in the total length of the hospital stay (median days, 55 (27,91) vs. 35 (18.5,81), *p* < 0.1). This is similar to the study by Wang et al. [36]. This might be due to the following factors: (1) The CCI, APACHE II score, Pitt bacteremia Score, and SOFA score, which reflect the severity of underlying diseases, did not show any difference between the two groups, and it might partially contribute a protective role in mortality in the current study. (2) Biological indicators were essentially the same between polymicrobial AB-BSI and monomicrobial AB-BSI (Table 2), meaning that there were no obvious differences in liver and kidney functions between these two groups. And, (3) there is no significant difference between the two in delayed antibiological therapy (9.7% vs. 7.7%, *p*=0.698), which might partially explain similar mortality between groups of polymicrobial AB-BSI and monomicrobial AB-BSI in the current study. Perhaps a higher study sample with sufficient statistical power is needed to demonstrate differences in polymicrobial and monomicrobial AB-BSI mortality.

## 5. Study Limitations

There are some limitations of this study. First, this is a single-center retrospective study, and although we collected data on AB-BSI from our hospital over four years, the number of patients is still relatively small. Second, because it is a retrospective study, we could only obtain information through electronic case records, resulting in some necessary information not being available; for example, we could only access the drug sensitivity information provided by the electronic case and could not perform an extended drug sensitivity test for MDR *A. baumannii*, much less provide data about the sequencing of *A. baumannii* isolates. Third, we are unable to control the variables such as the type and time of antibiotic use, which makes us unable to give corresponding suggestions on treatment. Therefore, a multicenter study with a large sample is necessary to further investigate the risk factors of polymicrobial AB-BSI for better prevention.

## 6. Conclusions

Polymicrobial AB-BSI accounted for a significant proportion among all AB-BSIs, and it did not influence mortality but was related to slightly longer total hospital stays. Multidrug resistance was associated with the development of polymicrobial AB-BSI but does not directly lead to polymicrobial AB-BSI, whereas resistance to piperacillin/tazobactam was highly correlated with polymicrobial AB-BSI. Therefore, while treating *A. baumannii* bloodstream infections, clinicians cannot ignore multidrug-resistant *A. baumannii*, especially piperacillin/tazobactam-resistant *A. baumannii*, which may predispose to the development of polymicrobial AB-BSI.

## Figures and Tables

**Table 1:** Baseline characteristics of patients with polymicrobial and monomicrobial AB-BSI.

Characteristics	Total (*n* = 204)	Monomicrobial AB-BSI (*n* = 165)	Polymicrobial AB-BSI (*n* = 39)	*p* value
Age, median years (IQR)	65.00 (49.25,76.75)	68.00 (53,77.50)	58 (40.00,76.00)	0.069

Male, *n* (%)	138 (67.6%)	111 (67.3%)	27 (69.2%)	0.814

Comorbidities, *n* (%)				
Diabetes mellitus	38 (18.6%)	33 (20.0%)	5 (12.8%)	0.300
Chronic kidney disease	22 (10.8%)	18 (10.9%)	4 (10.3%)	0.906
Chronic liver disease	25 (12.3%)	18 (10.9%)	7 (17.9%)	0.228
COPD or severe asthma	21 (10.3%)	18 (10.9%)	3 (7.7%)	0.552
Chronic cardiac insufficiency	33 (16.2%)	27 (16.4%)	6 (15.4%)	0.881
Hypertension	73 (35.8%)	59 (35.8%)	14 (35.9%)	0.987
Solid tumor	24 (11.8%)	21 (12.7%)	3 (7.7%)	0.380

Trauma	56 (27.5%)	37 (22.4%)	19 (48.7%)	0.001^*∗*^
Gastrointestinal hemorrhage	33 (16.2%)	24 (14.5%)	9 (23.1%)	0.193
Burn injury	35 (17.2%)	20 (12.1%)	15 (38.5%)	<0.001^*∗*^
Long-term corticoid treatment	43 (21.1%)	34 (20.6%)	9 (23.1%)	0.734
Cerebrovascular accident	34 (16.7%)	31 (18.8%)	3 (7.7%)	0.094

CCI, median (IQR)	3 (1.25,6)	4 (2,6)	2 (1,5)	0.077

APACHE II score, median (IQR)	14 (10,19.75)	14 (10,20)	14 (10,17)	0.482

SOFA score, median (IQR)	4 (2,7)	4 (2,7)	4 (2,7)	0.768

Pitt bacteremia score, median (IQR)	3 (1,4)	3 (1,4)	3 (2,4)	0.759

Hospitalization ward, *n* (%)				
ICU stay	96 (47.1%)	78 (47.3%)	18 (46.2%)	0.900

Previous treatment, *n* (%)				
Parenteral nutrition	106 (52%)	84 (50.9%)	22 (56.4%)	0.749
Mechanical ventilation	103 (50.5%)	82 (49.7%)	21 (53.8%)	0.641
Antibiotic exposure	175 (86.2%)	140 (85.4%)	35 (89.7%)	0.476

Surgery	73 (35.8%)	54 (32.7%)	19 (48.7%)	0.061
Renal replacement therapy	18 (8.8%)	15 (9.1%)	3 (7.7%)	0.396
Blood transfusion	91 (44.6%)	68 (41.2%)	23 (59%)	0.045^*∗*^

Invasive devices, *n* (%)				
Central line	168 (82.4%)	133 (80.6%)	35 (89.7%)	0.178
Indwelling urinary catheter	157 (77.0%)	122 (73.9%)	35 (89.7%)	0.035^*∗*^
Intraperitoneal drainage	28 (13.7%)	20 (12.1%)	8 (20.5%)	0.171

Prior hospital stays, median days (IQR)	13 (7,26)	12 (6,27)	16 (7,25)	0.327

Nosocomial infection, *n* (%)	124 (60.8%)	95 (57.6%)	29 (74.4%)	0.054

Delayed antibiotic therapy, *n* (%)	19 (9.3%)	16 (9.7%)	3 (7.7%)	0.698

Abbreviations: AB-BSI: *Acinetobacter baumannii* bloodstream infections; COPD: chronic obstructive pulmonary disease; CCI: Charlson Comorbidity Index; SOFA, sequential organ failure assessment; APACHE: acute physiology and chronic health; ICU: intensive care unit; IQR: interquartile range. ^∗^Significant.

**Table 2:** Comparison of biological indicators between the groups of polymicrobial and monomicrobial AB-BSI.

Biological indicators	Total (*n* = 204)	Monomicrobial AB-BSI (*n* = 165)	Polymicrobial AB-BSI (*n* = 39)	*p* value
Blood routine test				
WBC (×10^9^/L) (IQR)	10.4 (7.4,14.5)	10.5 (7.4,14.5)	10.1 (7.2,15.4)	0.833
Hematocrit (%) (IQR)	26.5 (22.3,31.9)	27.1 (22.3,32.4)	25 (22.3,30.8)	0.068
Platelet (×10^9^/L) (IQR)	170 (97,268.0)	167.5 (98.25,263.75)	182.5 (97,287.5)	0.632

Liver and kidney function				
Albumin (g/L) (mean ± S.D.)	29.0 ( 25.7,32.8)	31.09 ± 5.92	31.03 ± 5.15	0.177
GPT (U/L) (IQR)	30 (17,57)	30.5 (16,61)	32 (19.25,64.25)	0.688
GOT (U/L) (IQR)	38 (24.0,70.0)	38.5 (26.5,71.25)	36 (23,99)	0.952
ALP (IQR)	113 (84,165)	111.5 (80,167.25)	119 (96.25,160.25)	0.344
*γ*-GT (IQR)	77 (39,147)	73 (38.75,146.25)	86.5 (51.5,155.0)	0.177
LDH (IQR)	237 (168.75,333.75)	240 (169,323.75)	244 (176.25,348.75)	0.675
TBil (umol/L) (IQR)	14 (8.2,27.7)	13.7 (8.5,28.3)	14.75 (8.62,34.12)	0.693
SCr (umol/L) (IQR)	64 (49,93)	68 (53,105)	53 (41.5,85)	0.122
CRP (mg/L), median (IQR)	103 (64.5,162.0)	104.5 (73.45,162.0)	126.5 (73.12,235)	0.191

PCT (ng/ml), median (IQR)	1.31 (0.38,9.27)	1.5 (0.43,9.54)	0.95 (0.31,5.37)	0.235

Abbreviations: AB-BSI: *Acinetobacter baumannii* bloodstream infection; WBC: white blood count; GPT: glutamic-pyruvic transaminase; GOT: glutamic-oxaloacetic transaminase; ALP: alkaline phosphatase; *γ*-GT: gamma glutamyl transpeptidase; LDH: lactic dehydrogenase; TBil: total bilirubin; SCr: serum creatinine; CRP: C-reactive protein; PCT: procalcitonin; IQR: interquartile range.

**Table 3:** Comparison of the microbiological characteristics with monomicrobial AB-BSI and polymicrobial AB-BSI.

	Total (*n* = 204)	Monomicrobial AB-BSI (*n* = 165)	Polymicrobial AB-BSI (*n* = 39)	*p* value
Source of BSIs				
Pneumonia	63 (30.9%)	56 (33.9%)	7 (17.9%)	0.052
Skin and soft tissue infection	42 (20.6%)	27 (16.4%)	15 (38.5%)	0.002^*∗*^
Central venous catheter	30 (14.7%)	27 (16.4%)	3 (7.7%)	0.169
Intra-abdominal	44 (21.6%)	36 (21.8%)	8 (20.5%)	0.859
Primary BSI	21 (10.3%)	16 (9.7%)	5 (12.8%)	0.564
Bone and joint	1 (0.5%)	1 (0.6%)	0 (0%)	0.626
Urinary tract infection	1 (1.0%)	1 (0.6%)	1 (2.6%)	0.264
Antibiotic resistance^a^				
Cefoperazone/Sulbactam (127 vs 34)^b^	39 (24.2%)	32 (25.2%)	7 (20.6%)	0.557
Ceftazidime (162 vs. 37)^b^	27 (13.6%)	20 (12.3%)	7 (18.9%)	0.292
Meropenem (163 vs. 36)^b^	124 (62.3%)	105 (64.4%)	19 (52.8%)	0.192
Imipenem (164 vs. 39)^b^	120 (59.9%)	90 (54.9%)	30 (76.9%)	0.012^*∗*^
Ceftriaxone (120 vs. 32)^b^	113 (74.3%)	85 (70.8%)	28 (87.5%)	0.055
Cefepime (163 vs. 39)^b^	108 (53.5%)	79 (48.5%)	29 (74.4%)	0.004^*∗*^
Tigecycline (162 vs. 37)^b^	6 (3.0%)	5 (3.1%)	1 (2.7%)	0.902
Tobramycin (162 vs. 36)^b^	84 (42.4%)	63 (39.8%)	21 (58.3%)	0.033^*∗*^
Amikacin (107 vs. 27)^b^	32 (23.9%)	26 (24.3%)	6 (22.2%)	0.821
Gentamicin (116 vs. 29)^b^	79 (54.5%)	59 (50.9%)	20 (69.0%)	0.080
Piperacillin/Tazobactam (119 vs. 31)^b^	93 (62%)	67 (56.3%)	26 (83.9%)	0.005^*∗*^
Levofloxacin (165 vs. 39)^b^	68 (33.3%)	50 (30.3%)	18 (46.2%)	0.059
Ciprofloxacin (161 vs. 36)^b^	112 (56.9%)	85 (52.8%)	27 (75%)	0.015^*∗*^
Doxycycline (32 vs. 5)^b^	14 (37.8%)	12 (37.5%)	2 (40%)	0.915
Minocycline (116 vs. 31)^b^	124 (84.4%)	100 (86.2%)	24 (77.4%)	0.232
Ampicillin/Sulbactam (80 vs. 20)^b^	59 (59%)	51 (63.7%)	8 (40%)	0.053
MDR	160 ( 78.4%)	125 (75.8%)	35 (89.7%)	0.056

^a^Not all agents listed tested in all isolates. ^b^The numbers in parentheses represent the total numbers of *Acinetobacter Baumannii* isolates that performed the susceptibility test. Abbreviations: AB-BSI: *Acinetobacter Baumannii* bloodstream infection; BSI: bloodstream infection; MDR: multidrug resistance. ^*∗*^Significant.

**Table 4:** Multivariable logistic regression of factors associated with polymicrobial AB-BSI.

Variable	Unadjusted OR (95%CI)	*p* value	Adjusted OR (95%CI)	*p* value
Co-morbidities				
Trauma	3.27 (1.59,6.78)	0.001	2.348 (0.771,7.150)	0.133
Burn injury	4.30 (1.91,9.68)	0.000	3.536 (0.411,30.458)	0.250
Previous treatment				
Blood transfusion	2.05 (1.01,4.17)	0.047	0.593 (0.206,1.706)	0.333
Indwelling urinary catheter	3.08 (1.04,9.19)	0.043	1.722 (0.360,8.244)	0.497
Source of bloodstream infections				
Skin and soft tissue infection	3.19 (1.49,6.87)	0.003	0.790 (0.102,6.136)	0.822
Antibiotic resistance				
Imipenem	2.74 (1.22,6.14)	0.014	0.019 (0.000,1.886)	0.091
Cefepime	3.08 (1.41,6.74)	0.005	2.234 (0.231,21.571)	0.487
Tobramycin	2.20 (1.06,4.58)	0.035	1.007 (0.288,3.528)	0.991
Piperacillin/Tazobactam	4.04 (1.45,11.23)	0.008	14.48 (2.07,101.24)	0.007^*∗*^
Ciprofloxacin	2.68 (1.19,6.06)	0.018	4.995 (0.087,287.043)	0.436

Abbreviations: AB-BSI: *Acinetobacter Baumannii* bloodstream infection; OR: odds ratio; CI: confidence interval. ^*∗*^Significant.

**Table 5:** Comparison of outcome between monomicrobial and polymicrobial AB-BSI.

Prognostic indicators	Total (*n* = 204)	Monomicrobial AB-BSI (*n* = 165)	Polymicrobial AB-BSI (*n* = 39)	*p* value
Total hospitalization days (M) (IQR)	38.5 (20.25,83)	35 (18.5,81)	55 (27,91)	0.09
Total ICU residence days (M) (IQR)	23 (12,46)	23 (12,46)	22.5 (8.25,56.5)	0.71
Sepsis	148 (72.5%)	119 (72.1%)	29 (74.4%)	0.78
Cause septic shock (*n*, %)	22 (10.8%)	17 (10.3%)	5 (12.8%)	0.65
7-day total mortality rate (*n*, %)	36 (17.6%)	31 (18.8%)	5 (12.8%)	0.38
14-day total mortality rate (*n*, %)	45 (22.1%)	38 (23.0%)	7 (17.9%)	0.49
28-day total mortality rate (*n*, %)	61 (29.9%)	48 (29.1%)	13 (33.3%)	0.60
In-hospital mortality (*n*, %)	78 (38.4%)	61 (37.2%)	17 (43.6%)	0.46

Abbreviations: M: median; IQR: interquartile range; ICU: intensive care unit; AB-BSI: *Acinetobacter baumannii* bloodstream infections.

## Data Availability

All data generated or analyzed during this study are included in this manuscript.

## Abbreviations

AB-BSI: *Acinetobacter baumannii* bloodstream infections
BSI: Bloodstream infections
*A. baumannii*: *Acinetobacter baumannii*
MDR: Multidrug resistant
CLSI: Clinical and Laboratory Standards
CDC: Centers for Disease Control and Prevention
SOFA: Sequential organ failure assessment
CCI: Charlson Comorbidity Index
APACHE: Acute physiology and chronic health evaluation
IQR: Interquartile range
OR: Odds ratio
CI: Confidence interval
*S. aureus*: *Staphylococcus aureus*
*E. faecium*: *Enterococcus faecium*
MRSA: Methicillin-resistant *S aureus*
ICU: Intensive care unit.

## Supplementary Materials

Supplementary Materials Supplementary Figure 1: Distribution of multidrug-resistant *Acinetobacter baumannii* and polymicrobial bloodstream infections over the years.
