# Supplementary material for: Multidrug-Resistant Acinetobacter baumannii May Cause Patients to Develop Polymicrobial Bloodstream Infection
Source: Can J Infect Dis Med Microbiol. 2022 Jun 24;2022:8368578. doi: 10.1155/2022/8368578 (PMC9249487; doi:10.1155/2022/8368578)
Supplement: Supplementary Materials — Supplementary Figure 1: Distribution of multidrug-resistant Acinetobacter baumannii and polymicrobial bloodstream infections over the years. [file 8368578.f1.pdf]

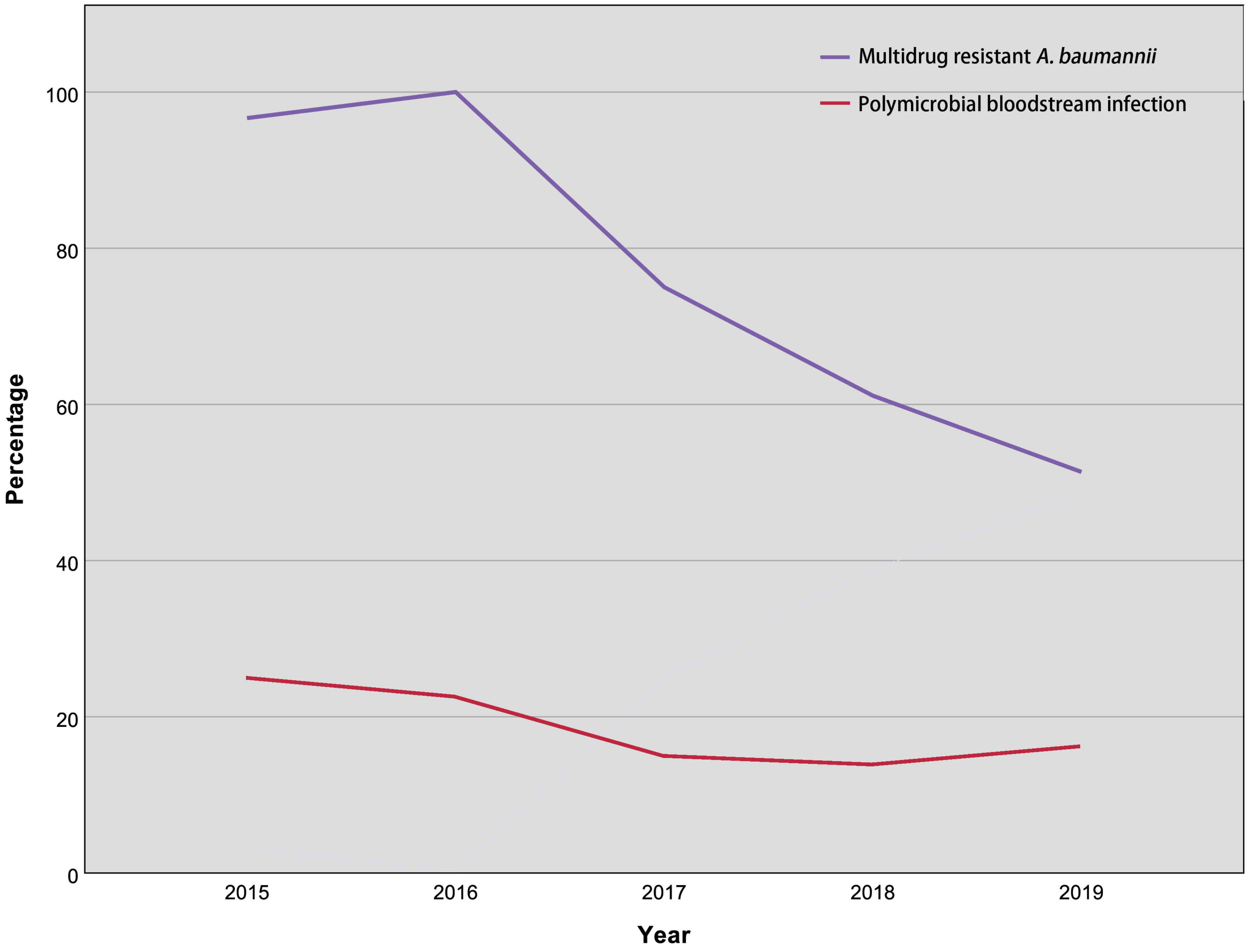

Supplementary Figure 1: Distribution of multi-drug resistant *Acinetobacter baumannii* and polymicrobial bloodstream infections over the years
